# Human Cytomegalovirus Infection of Epithelial Cells Increases SARS-CoV-2 Superinfection by Upregulating the ACE2 Receptor

**DOI:** 10.1093/infdis/jiac452

**Published:** 2022-11-21

**Authors:** Marianne R Perera, Edward J D Greenwood, Thomas W M Crozier, Elizabeth G Elder, Janika Schmitt, Colin M Crump, Paul J Lehner, Mark R Wills, John H Sinclair, the Cambridge Institute of Therapeutic Immunology and Infectious Disease-National Institute of Health Research COVID BioResource Collaboration, Stephen Baker, John Bradley, Gordon Dougan, Christoph Hess, Ian Goodfellow, Ravi Gupta, Nathalie Kingston, Paul J Lehner, Paul A Lyons, Nicholas J Matheson, Willem H Owehand, Caroline Saunders, Kenneth G C Smith, Charlotte Summers, James E D Thaventhiran, Mark Toshner, Michael P Weekes, Ashlea Bucke, Jo Calder, Laura Canna, Jason Domingo, Anne Elmer, Stewart Fuller, Julie Harris, Sarah Hewitt, Jane Kennet, Sherly Jose, Jenny Kourampa, Anne Meadows, Criona O’Brien, Jane Price, Cherry Publico, Rebecca Rastall, Carla Ribeiro, Jane Rowlands, Valentina Ruffolo, Hugo Tordesillas, Ben Bullman, Benjamin J Dunmore, Stuart Fawke, Stefan Gräf, Josh Hodgson, Christopher Huang, Kelvin Hunter, Emma Jones, Ekaterina Legchenko, Cecilia Matara, Jennifer Martin, Ciara O’Donnell, Linda Pointon, Nicole Pond, Joy Shih, Rachel Sutcliffe, Tobias Tilly, Carmen Treacy, Zhen Tong, Jennifer Wood, Marta Wylot, Laura Bergamaschi, Ariana Betancourt, Georgie Bower, Aloka De Sa, Madeline Epping, Stuart Fawke, Oisin Huhn, Sarah Jackson, Isobel Jarvis, Jimmy Marsden, Francesca Nice, Georgina Okecha, Ommar Omarjee, Marianne Perera, Nathan Richoz, Rahul Sharma, Lori Turner, Eckart M D D De Bie, Katherine Bunclark, Masa Josipovic, Michael Mackay, Federica Mescia, Alice Michael, Sabrina Rossi, Mayurun Selvan, Sarah Spencer, Cissy Yong, Ali Ansaripour, Alice Michael, Lucy Mwaura, Caroline Patterson, Gary Polwarth, Petra Polgarova, Giovanni di Stefano, John Allison, Heather Biggs, Helen Butcher, Daniela Caputo, Matt Chandler, Patrick F Chinnery, Debbie Clapham-Riley, Anne-Maree Dean, Eleanor Dewhurst, Christian Fernandez, Anita Furlong, Anne George, Barbara Graves, Jennifer Gray, Sabine Hein, Tasmin Ivers, Mary Kasanicki, Emma Le Gresley, Rachel Linger, Sarah Meloy, Alexei Moulton, Francesca Muldoon, Nigel Ovington, Sofia Papadia, Roxana Paraschiv, Christopher Penkett, Isabel Phelan, Venkatesh Ranganath, Jennifer Sambrook, Katherine Schon, Hannah Stark, Kathleen E Stirrups, Paul Townsend, Julie von Ziegenweidt, Neil Walker, Jennifer Webster

**Affiliations:** Department of Medicine, Cambridge Institute of Therapeutic Immunology and Infectious Disease, University of Cambridge, Addenbrooke's Hospital, Cambridge, United Kingdom; Department of Medicine, Cambridge Institute of Therapeutic Immunology and Infectious Disease, University of Cambridge, Addenbrooke's Hospital, Cambridge, United Kingdom; Department of Medicine, Cambridge Institute of Therapeutic Immunology and Infectious Disease, University of Cambridge, Addenbrooke's Hospital, Cambridge, United Kingdom; Department of Microbiology, National Veterinary Institute Uppsala, Sweden; Department of Medicine, Cambridge Institute of Therapeutic Immunology and Infectious Disease, University of Cambridge, Addenbrooke's Hospital, Cambridge, United Kingdom; Department of Pathology, University of Cambridge, Cambridge, United Kingdom; Department of Medicine, Cambridge Institute of Therapeutic Immunology and Infectious Disease, University of Cambridge, Addenbrooke's Hospital, Cambridge, United Kingdom; Department of Medicine, Cambridge Institute of Therapeutic Immunology and Infectious Disease, University of Cambridge, Addenbrooke's Hospital, Cambridge, United Kingdom; Department of Medicine, Cambridge Institute of Therapeutic Immunology and Infectious Disease, University of Cambridge, Addenbrooke's Hospital, Cambridge, United Kingdom

**Keywords:** ACE2, COVID-19, HCMV, SARS-CoV-2, coinfection, human cytomegalovirus

## Abstract

Severe acute respiratory syndrome coronavirus 2 (SARS-CoV-2), the causative agent of coronavirus disease 2019 (COVID-19), has caused widespread morbidity and mortality since its onset in late 2019. Here, we demonstrate that prior infection with human cytomegalovirus (HCMV) substantially increases infection with SARS-CoV-2 in vitro. HCMV is a common herpesvirus carried by 40%–100% of the population, which can reactivate in the lung under inflammatory conditions, such as those resulting from SARS-CoV-2 infection. We show in both endothelial and epithelial cell types that HCMV infection upregulates ACE2, the SARS-CoV-2 cell entry receptor. These observations suggest that HCMV reactivation events in the lung of healthy HCMV carriers could exacerbate SARS-CoV-2 infection and subsequent COVID-19 symptoms. This effect could contribute to the disparity of disease severity seen in ethnic minorities and those with lower socioeconomic status, due to their higher CMV seroprevalence. Our results warrant further clinical investigation as to whether HCMV infection influences the pathogenesis of SARS-CoV-2.

## Teaser

Human cytomegalovirus (HCMV), a widespread pathogen, upregulates ACE2 in vitro in epithelial cells. This results in increased SARS-CoV-2 infection. We, therefore, propose that HCMV (carried by up to 90% in some populations) may play an important role in COVID-19 severity.

The severity of severe acute respiratory syndrome coronavirus 2 (SARS-CoV-2) infection varies wildly between individuals [1]. The reasons for this are unclear but are likely to be complex and multifactorial. One important factor is coinfection with another pathogen, which may exacerbate existing tissue damage [2] or may even drive higher SARS-CoV-2 replication [3]. Interestingly, the inflammation accompanying SARS-CoV-2 infections has the potential to reactivate the latent form of an extremely common virus, human cytomegalovirus (HCMV), which has led to the suggestion that HCMV infection could influence the course of coronavirus disease 2019 (COVID-19) [4–6].

Clinical reactivation of HCMV occurs under inflammatory conditions and during critical illness and pneumonia [7]. Proinflammatory cytokines such as interleukin 6 (IL-6), tumor necrosis factor-α (TNF-α) and IL-1β increase reactivation events of latent HCMV. Perhaps unsurprisingly, there have been several reports of HCMV reactivation in severe cases of COVID-19 [8–15], a disease in which hyperinflammation and pneumonia are common features.

In a number of clinical scenarios, HCMV often reactivates in the lung and bowel [16, 17], organs that are also infected by SARS-CoV-2 [18–20]. Following reactivation, HCMV extensively modifies the host cell and its environment. Interestingly, hospitalized COVID-19 patients are significantly more likely to be seropositive for HCMV than nonhospitalized patients [21, 22], and reactivation of HCMV during severe COVID-19 correlates with prolonged mechanical ventilation [12].

Here, we investigated whether preinfection with HCMV affects the outcome of SARS-CoV-2 superinfection. We find that HCMV infection of a number of biologically relevant cell types upregulates the SARS-CoV-2 entry receptor, angiotensin converting enzyme 2 (ACE2), and significantly enhances SARS-CoV-2 infection, which could have important implications for the pathogenesis and treatment of COVID-19.

## METHODS

The study was conducted according to the guidelines of the Declaration of Helsinki, and approved by the Institutional Review Board (or Ethics Committee) of Health Research Authority Cambridge Central Research Ethics Committee (REC reference 97/092).

### Cells

All cells were cultured at 37°C in 5% CO_2_. Human umbilical vein endothelial cells (HUVECs) were cultured in Endothelial Cell Growth Medium 2 (C-22111; PromoCell) treated with the provided supplements according to the manufacturer's instructions. Caco-2 cells were cultured in Eagle's Minimum Essential Medium (Sigma), supplemented with 20% fetal bovine serum (FBS). Retinal pigment epithelial cells (RPE-1s, ATCC CRL-4000) were maintained in Dulbecco's Modified Eagle Medium (Sigma) supplemented with 10% heat-inactivated FBS (PAN Biotech), 100 U/mL penicillin, and 100 µg/mL streptomycin (Sigma).

### Viruses

All HCMV viruses used in this study were recombinant strains of HCMV based on TB40E BAC4 strains. Cells were infected at a multiplicity of infection (MOI) of 1 as based on titration on RPE-1 cells.

ZsGreen-tagged SARS-CoV-2 virus was derived from the plasmid, pCCI-4K-SARS-CoV-2-ZsGreen, a kind gift from Professor Sam Wilson, University of Glasgow and is described in Rihn et al [23]. The percentage of cells infected with SARS-CoV-2 was quantified by averaging the number of ZsGreen-positive cells in 3 random fields of view per replicate as performed in Chu et al [24].

A recombinant herpes simplex virus-1 (HSV-1) encoding eYFP-tagged ICP0 was constructed in a bacterial artificial chromosome (BAC) cloned HSV-1 strain KOS. The coding sequence for eYFP containing the A206K mutation (to inhibit eYFP dimerization) was inserted at the 5′ end of exon 1 of both copies of the RL2 gene in the KOS BAC, in frame with the ICP0 open reading frame, using primers COL585 and COL586 (COL585: CCC CCA GGG ACC CTC CGT CAG CGA CCC TCC AGC CGC ATA CGA CCC CCA TGG TGA GCA AGG GCG AGG AG; COL586: CGC TGG GGG CGG CCC TCA GGC CGG GTA CTC GCT CCG GGG CGG GGC TCC TTG TAC AGC TCG TCC ATG) and the 2-step Red recombination method. ΔgE HSV-1 is described in Albecka et al [25].

### Cytomegalovirus IgG ELISA

Blood samples were collected from patients and health care workers who had tested polymerase chain reaction (PCR) positive for SARS-CoV-2 from Cambridge University Hospitals between March and July 2020. Informed consent was obtained from all subjects involved in the study.

Serum was separated from whole blood by centrifugation. Serum was tested for HCMV immunoglobulin G (IgG) levels with Captia Cytomegalovirus (CMV) IgG Enzyme-Linked Immunosorbent Assay (ELISA) from Trinity Biotech according to the manufacturer's instructions.

### Immunofluorescence

SARS-CoV-2–infected cells were fixed in 4% paraformaldehyde (PFA). All other cells were fixed in 2% PFA. Cells were permeabilized with 0.1% Triton X-100. Cells stained for ACE2 were blocked with normal rabbit IgG. Primary antibody was added at a 1:100 dilution for 1 hour and subsequently washed off 3 times in Tris-buffered saline (TBS). Secondary antibodies (goat anti-mouse Alexa Fluor 594 [A11005; Thermo Fisher], goat anti-rabbit Alexa Fluor 594 [A21207; ThermoFisher], or goat anti-rabbit Alexa Fluor 488 [ab150077]) were diluted 1:500 and incubated with Hoeschst 33342 nuclear stain (1μg/mL; Sigma) for 1 hour at room temperature, before 3 washes with TBS. Cells were imaged with a widefield Nikon TE200 microscope and all images were processed with ImageJ software.

### Quantitative Real-Time Polymerase Chain Reaction

Cells were harvested in TRIzol reagent (Invitrogen) and RNA was extracted with a Qiagen RNeasy kit. Contaminating DNA was eliminated using gDNA Wipeout Buffer (Qiagen) and RNA was reverse transcribed with a QuantiTect reverse transcription kit. Levels of RNA were assessed by quantitative real-time PCR (RTqPCR), using the following primers: ACE2 forward: GGA CCC AGG AAA TGT TCA GA; ACE2 reverse: GGC TGC AGA AAG TGA CAT GA; HCMV IE forward: GGA CCC TGT AAT CCT GAC G; HCMV IE reverse: ATC TTT CTC GGG GTT CTC GT; 18S forward: GTA ACC CGT TGA ACC CCA; 18S reverse: CCA TCC AAT CGG TAG CG.

### Sodium Dodecyl Sulfate Polyacrylamide Gel Electrophoresis and Immunoblotting

Samples were lysed with Laemmli buffer directly and were run on 10% sodium dodecyl sulfate (SDS)-polyacrylamide gels with a 5% stacking gel. Proteins were then transferred overnight at 20 V to Hybond nitrocellulose membranes (Amersham Biosciences), which were then blocked for 1 hour in 5% w/v skim milk dissolved in TBS (5% milk-TBS) at room temperature, followed by overnight incubation at 4°C with the following primary antibodies diluted in 5% milk-TBS: α-ACE2 (ab108209, 1:500; Abcam), α-TMPRSS2 (ab242384, 1:1000; Abcam), α-actin, (ab8227, 1:2000; Abcam), α-HCMV IE1/2 (11-003, 1:1000; Argene), and α-HSV-1 ICP0 (mouse monoclonal antibody hybridoma supernatant, 11060, 1:5). Blots were washed 3 times in TBS with 0.5% Tween20 (TBS-Tween), and then incubated with secondary antibodies conjugated to horseradish peroxidase. Blots were washed 3 times in TBS-Tween and then incubated with Amersham ECL Western Blotting detection reagent (GE Healthcare Life Sciences) for 5 minutes before being exposed to autoradiography film (Fujifilm).

## RESULTS

### Severity of COVID-19 Cases Correlates With HCMV Serostatus and Levels of CMV IgG

As other pathogens have been shown to influence the outcome of COVID-19 [2, 3], we tested whether there was any link between HCMV serostatus and disease severity in SARS-CoV-2–infected individuals. Serum was collected from a cohort of patients and health care workers who had tested PCR positive for SARS-CoV-2 from Cambridge University Hospitals between March and July 2020 (details of these have been previously described [26]). These patients were stratified into “not hospitalized” (which comprised the asymptomatic or mildly ill) or “hospitalized with oxygen” (patients who were hospitalized and required supplemental oxygen or assisted ventilation). We tested the HCMV serostatus of these patients by assaying their sera for the presence of HCMV-specific IgG (indicating previous exposure to HCMV).

**Figure 1.**
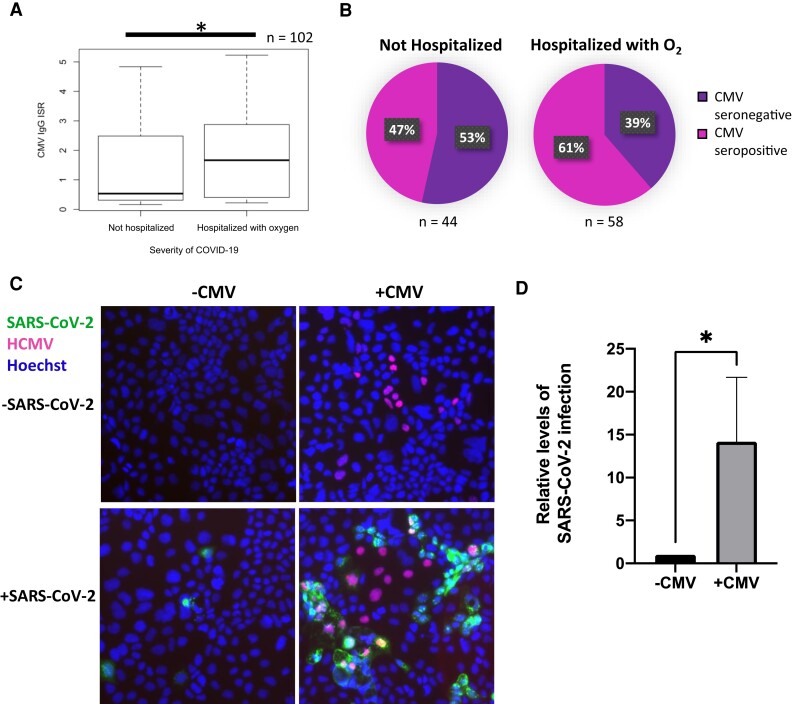
Prior infection with HCMV increases SARS-CoV-2 superinfection in vitro. *A*, Levels of HCMV IgG in patients with PCR-confirmed SARS-CoV-2 infection were measured by ELISA, and the ISR determined (which reflects the levels of HCMV IgG). Patients were stratified into not hospitalized (asymptomatic or mild infection) or hospitalized with O_2_ (hospitalized with supplemental O_2_ or hospitalized with assisted ventilation). Box and whisker plots show ISR distributions for these 2 groups; n = 102. Significant difference was determined using a two-tailed Mann Whitney U test. *P < .05. *B*, Immune response ratio from (*A*) was used to class patients as CMV seropositive or seronegative, and the proportion of patients who were not hospitalized or hospitalized with oxygen that were CMV seropositive or CMV seronegative is illustrated in a pie chart. *C* and *D*, Caco-2 epithelial cells were infected with HCMV for 4 days before being infected with ZsGreen-tagged SARS-CoV-2 at a multiplicity of infection of 0.01. *C*, Two days postinfection with SARS-CoV-2, cells were fixed, stained for HCMV IE1/2 (red), and stained with Hoechst (blue) and imaged on a fluorescence microscope. *D*, Graph shows mean percent SARS-CoV-2 infection from 3 randomly chosen images for each of 4 replicates over 2 independent repeats. Error bars show standard deviation. Statistical significance was determined using a 2-tailed Mann-Whitney *U* test, *P < .05. Abbreviations: ELISA, enzyme-linked immunosorbent assay; HCMV, human cytomegalovirus; IgG, immunoglobulin G; ISR, immune status ratio; PCR, polymerase chain reaction; SARS-CoV-2, polymerase chain reaction.

Interestingly, we found significantly higher levels of HCMV-specific IgG in patients that displayed severe illness (*P* < .05; Figure 1*A*) and that HCMV-seropositive individuals accounted for a higher proportion of the severe cases of COVID-19 (Figure 1*B*). Increased HCMV seroprevalence amongst the more severe cases of COVID-19 has also been demonstrated by others [21, 22].

### Prior Infection With HCMV Significantly Increases the Number of Cells Infected by Live SARS-CoV-2 Virus

HCMV routinely reactivates in the lung and bowel, organs targeted by SARS-CoV-2. As HCMV modulates its cellular environment, we speculated that the association between HCMV seropositivity and COVID-19 severity detailed in Figure 1*A* and 1*B* might be causal, and that coinfection with HCMV promotes SARS-CoV-2 infection. To investigate this, we used live SARS-CoV-2 virus to infect HCMV-infected Caco-2, a cell line which supports both SARS-CoV-2 replication and HCMV replication (Supplementary Figure 1). Four days postinfection (dpi) with HCMV, cells were superinfected with ZsGreen-tagged SARS-CoV-2 for 2 further days, before being fixed and stained for HCMV immediate early (IE) protein. Intriguingly, we found that preinfection with HCMV caused an approximately 14-fold increase in the number of cells infected with SARS-CoV-2 (Figure 1*C* and 1*D*).

### HCMV Upregulates the SARS-CoV-2 Receptor, ACE2

We next wanted to determine how lytic HCMV infection renders cells more susceptible to SARS-CoV-2 infection. Entry of SARS-CoV-2 into cells is mediated by the spike protein, (S), which is located in the viral envelope and binds to the cell surface ACE2 receptor. The tissue distribution pattern of ACE2 therefore reflects the tropism of SARS-CoV-2. Efficient SARS-CoV-2 entry also requires the cellular serine protease TMPRSS2, which cleaves the spike protein, allowing fusion of viral and cellular membranes to occur.

**Figure 2.**
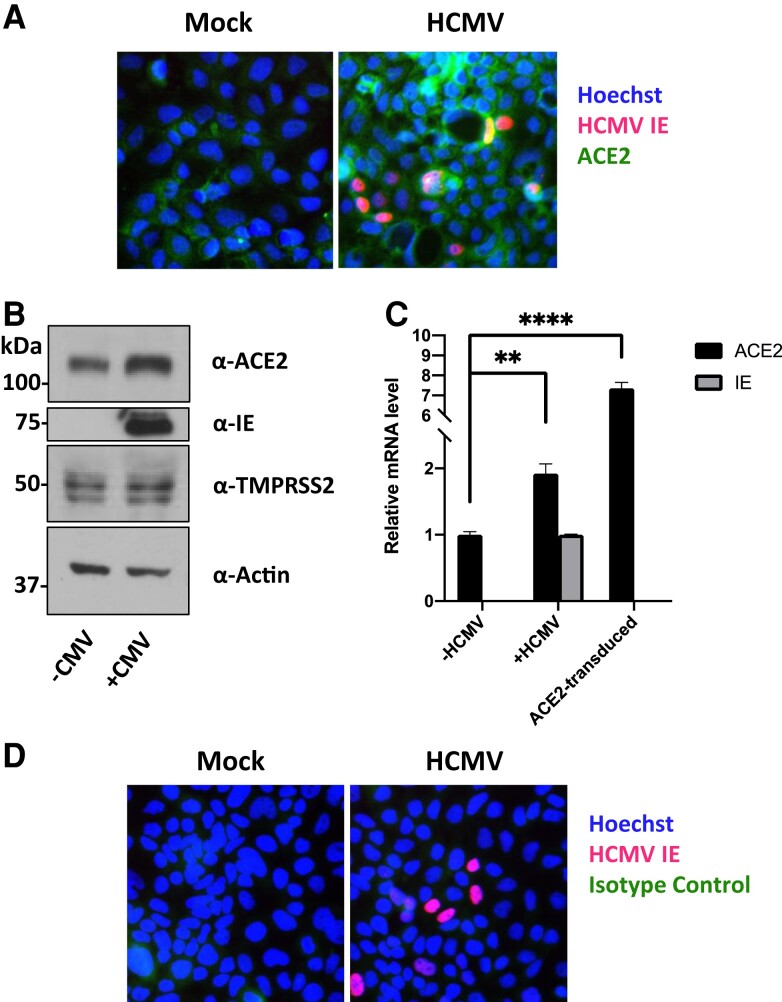
HCMV infection upregulates ACE2 on Caco-2 epithelial cells. Caco-2 cells were infected with HCMV at a multiplicity of infection of 0.1. *A*, Four dpi, cells were fixed and stained with an anti-HCMV IE1/2 antibody (red), Hoechst stain (blue), and α-ACE2 antibody (green). *B*, Four dpi, protein lysates from cells were harvested and assessed for levels of ACE2, TMPRSS2, HCMV IE1/2, and the loading control actin by immunoblot. *C*, Four dpi, RNA was harvested from cells and levels of ACE2, HCMV IE, and the housekeeping RNA, 18S, were measured by RTqPCR. As a positive control, RNA was also harvested from HeLa cells transduced to overexpress ACE2. Significance was determined using a 1-way ANOVA with Dunnett post hoc testing. Error bars show standard deviation. *D*, As in (*A*), but cells were stained with an isotype control antibody (green). Abbreviations: ACE2, angiotensin converting enzyme 2; dpi, days postinfection; HCMV, human cytomegalovirus; IE, immediate early protein; RTqPCR, quantitative real-time polymerase chain reaction.

We therefore tested whether HCMV induces changes in ACE2 and/or TMPRSS2 levels. We infected Caco-2 cells with HCMV and 4 dpi, either fixed cells and stained for ACE2 by immunofluorescence (Figure 2*A*) or harvested protein and blotted for ACE2 and TMPRSS2 (Figure 2*B*). Following HCMV infection we observed a striking upregulation of ACE2 protein, with a small increase in TMPRSS2, in both immunofluorescence and immunoblot assays. We also measured levels of ACE2 RNA and found that this was almost doubled by HCMV infection (Figure 2*C*). As HCMV encodes Fcγ receptors as well as upregulating cellular Fcγ receptors on some cells, we also included isotype-matched controls to rule out nonspecific staining (Figure 2*D*).

**Figure 3.**
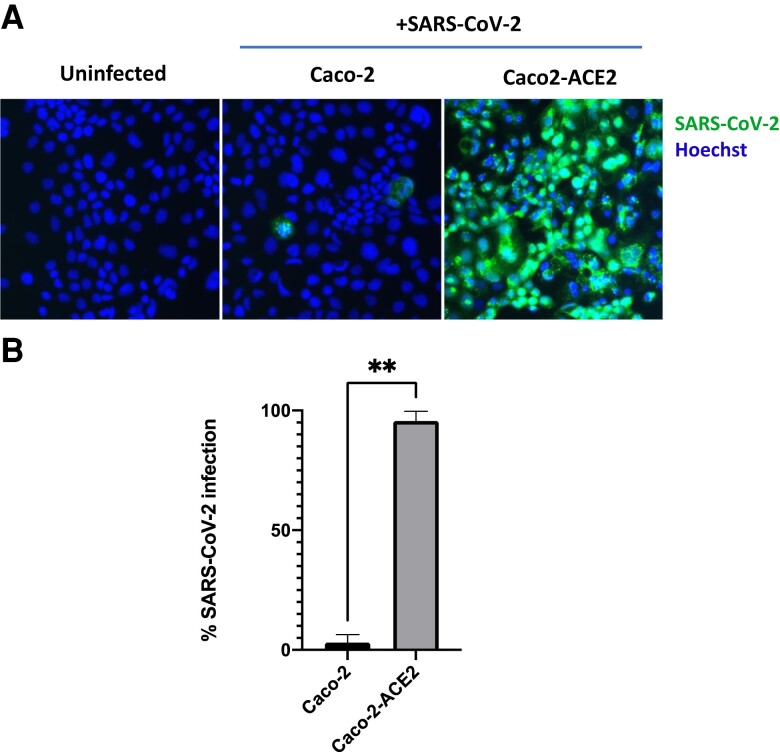
Overexpression of ACE2 increases SARS-CoV-2 infection rate in Caco-2 cells. Untransduced Caco-2 cells or Caco-2 cells transduced with an ACE2-overexpression lentivirus were infected with ZsGreen-tagged SARS-CoV-2 virus at a low multiplicity of infection (0.01). *A*, Two days postinfection, cells were fixed, Hoechst stained, and imaged with a fluorescence microscope. *B*, Quantification of results shown in (*A*), counted from 6 random fields of view over 2 independent repeats. Error bars show standard deviation. Statistical significance was determined by a 2-tailed Mann-Whitney *U* test. **P < .01. Abbreviations: ACE2, angiotensin converting enzyme 2; SARS-CoV-2, polymerase chain reaction.

To ensure that increased levels of ACE2 would indeed result in increased SARS-CoV-2 infection, we also transduced Caco-2 cells with an ACE2 overexpression lentivirus and infected these cells with SARS-CoV-2. Transduction with ACE2 resulted in almost all cells becoming permissive to SARS-CoV-2 infection (Figure 3). This confirms that ACE2 expression is a limiting factor in the permissiveness of Caco-2 cells to SARS-CoV-2.

### HCMV Upregulates ACE2 in Different Cell Types

**Figure 4.**
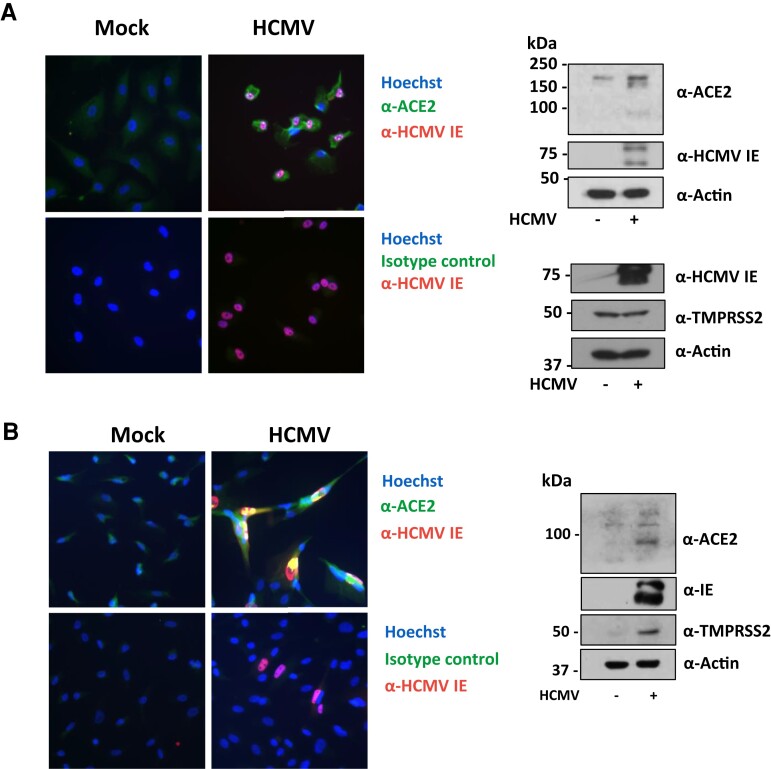
HCMV infection upregulates ACE2 on epithelial and endothelial cells. *A*, Retinal pigment epithelial cells or (*B*) human umbilical vein endothelial cells were infected with HCMV at a multiplicity of infection of 1 or 0.1, respectively. *A* and *B*, (left) Four dpi, cells were fixed and stained with an anti-HCMV IE1/2 antibody (red), Hoechst stain (blue), and either an α-ACE2 antibody or its isotype control (green). *A* and *B*, (right) Four dpi, protein lysates from cells were harvested and assessed for levels of ACE2 or TMPRSS2, HCMV IE1/2, and the loading control actin by immunoblot. Abbreviations: ACE2, angiotensin converting enzyme 2; dpi, days postinfection; HCMV, human cytomegalovirus; IE, immediate early protein.

We also tested whether HCMV could upregulate ACE2 in other cell types commonly infected by HCMV. We found that RPEs had barely detectable amounts of ACE2, but this increased upon HCMV infection as measured by both indirect immunofluorescence and western blot analysis (Figure 4*A*). This raises the possibility that HCMV infection could increase the range of SARS-CoV-2–infectable cells.

While epithelial cells in the respiratory tract are key entry sites for SARS-CoV-2 infection, endothelial cells are thought to be important players in COVID-19 pathology due to their central role in regulating inflammation and thrombosis [27–29]. Therefore, we also tested whether HCMV-mediated ACE2 upregulation occurs in endothelial cells, using HUVEC, a primary endothelial cell type commonly used in HCMV infection models. Results in Figure 4*B* show that uninfected HUVECs demonstrated little expression of either ACE2 or TMPRSS2. However, HCMV infection clearly led to upregulation of ACE2 and substantial upregulation of TMPRSS2 when analyzed by both indirect immunofluorescence and western blot (Figure 4*B*).

### Upregulation of ACE2 by HCMV May Involve a Soluble Factor Released From Infected Cells

**Figure 5.**
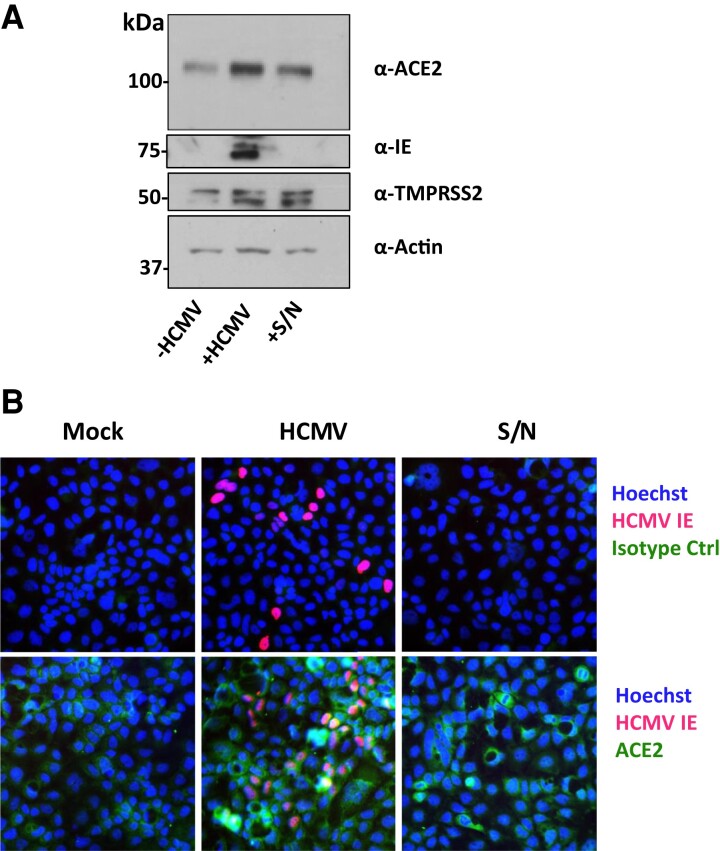
Supernatant from HCMV-infected cells upregulates ACE2 on uninfected Caco-2 cells. Caco-2 cells were mock infected (−HCMV) or infected (+HCMV) with HCMV at a multiplicity of infection of 0.1. Every 24 hours, supernatant from HCMV infected cells was transferred to another well of uninfected Caco-2 cells (+S/N). *A*, Protein lysates from these cells were then immunoblotted for ACE2, TMPRSS2, HCMV IE1/2, and the loading control actin. *B*, Four days postinfection, cells were fixed and stained with anti-HCMV IE1/2 antibody (red), Hoechst stain (blue), and either α-ACE2 antibody or its isotype control (green). Abbreviations: ACE2, angiotensin converting enzyme 2; HCMV, human cytomegalovirus; IE, immediate early protein; S/N, supernatant.

In Caco-2 cells infected with HCMV, many uninfected, bystander cells also upregulated ACE2 (Figure 2*A*) and, indeed, SARS-CoV-2 superinfection occurred predominantly in uninfected Caco-2 cells surrounding HCMV-infected Caco-2 cells (Figure 1*C*). We therefore tested whether a secreted factor from HCMV-infected cells could be responsible for upregulating ACE2 on bystander cells. To do this, we infected Caco-2 cells with HCMV and 24 hours postinfection (hpi) harvested supernatants from these cells and transferred them to uninfected Caco-2 cells. Fresh supernatant was transferred each day for 4 days, after which cells were either harvested and lysed for immunoblot (Figure 5*A*) or stained for ACE2 by immunofluorescence (Figure 5*B*). We observed that uninfected Caco-2 cells treated with HCMV-derived supernatant also upregulated ACE2, suggesting the involvement of an HCMV-induced secreted factor in ACE2 upregulation.

Meta-analysis studies have analyzed the expression pattern of ACE2 and suggested that ACE2 could be an interferon stimulated gene that can be upregulated by infection with other viruses [30–32]. A number of these studies also showed that, in vitro, ACE2 mRNA levels can increase with interferon treatment or treatment of cells with interferon-inducing insults [31–33], although this did appear to be restricted to certain cell types [33].

**Figure 6.**
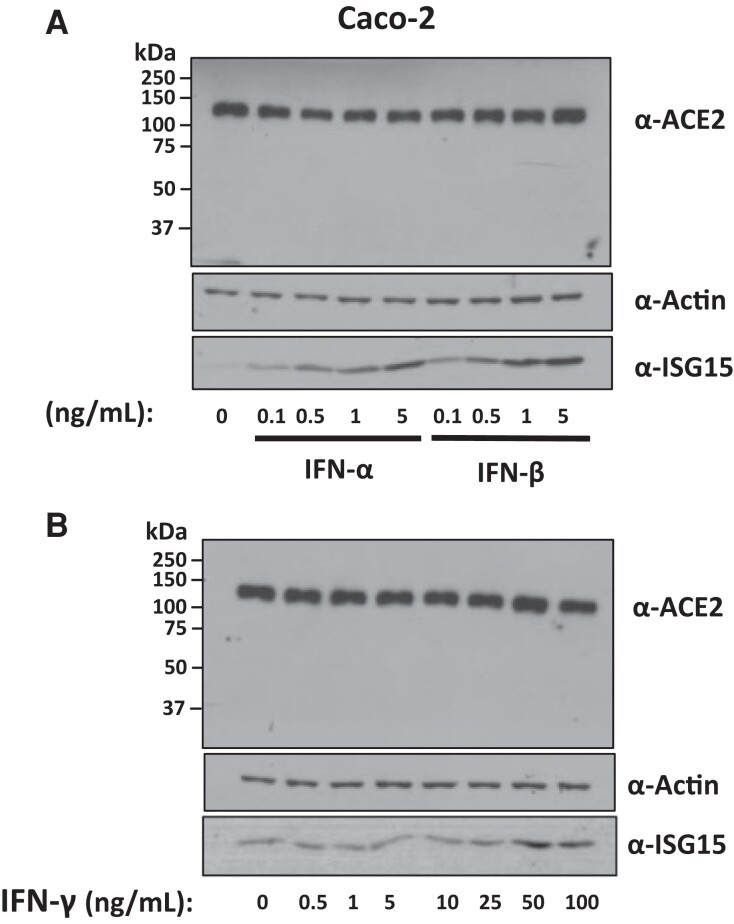
Exogenous IFNs do not increase levels of ACE2 in Caco-2 cells. Caco-2 cells were treated with increasing concentrations of (*A*) IFN-α or IFN-β or (*B*) IFN-γ. Four days posttreatment, protein was harvested form these cells and assayed for levels of ACE2, the loading control actin, or the positive control ISG15. Abbreviations: ACE2, angiotensin converting enzyme 2; IFN, interferon; ISG15, interferon-stimulated gene 15.

To test the possibility that interferon (IFN) secreted by HCMV-infected cells could be responsible for augmenting ACE2 levels in our experiments, we treated Caco-2 cells with different concentrations of IFN-α, -β, and -γ. As shown in Figure 6, Caco-2 cells treated with varying concentrations of IFN-α, -β (Figure 6*A*), or -γ (Figure 6*B*) showed no increase in ACE2 protein expression. This mirrors the findings of Bai et al, who found that influenza virus promoted SARS-CoV-2 infection and robustly upregulated ACE2 in A549 cells, but treatment with exogenous IFN-α had no significant effect on ACE2 mRNA levels in these cells [3].

Interestingly, it has been reported that the increase in ACE2 levels driven by interferon corresponds only to a truncated isoform of ACE2, which cannot act as an entry receptor for SARS-CoV-2 [34–36]. For this reason, we blotted the entire nitrocellulose membrane for ACE2 to also detect any truncated ACE2. However, we observed no bands other than the prominent ACE2 band at roughly 120 kDa. It is possible that a truncated ACE2 isoform was below the limit of our detection, or that truncated ACE2 lacked the epitope targeted by the monoclonal antibody used.

### HSV-1 Does Not Promote SARS-CoV-2 Superinfection

After observing that HCMV promoted SARS-CoV-2 infection, likely via upregulation of ACE2, we questioned whether other herpesviruses might also similarly affect SARS-CoV-2 infection. HSV-1 is a member of the alphaherpesvirus subfamily. As a herpesvirus, it also undergoes lifelong latent infection in its host, from which it reactivates periodically, and can infect a range of tissue types. HSV-1 is also an extremely widespread pathogen, with a prevalence of around 67% in people younger than 50 years across the globe.

**Figure 7.**
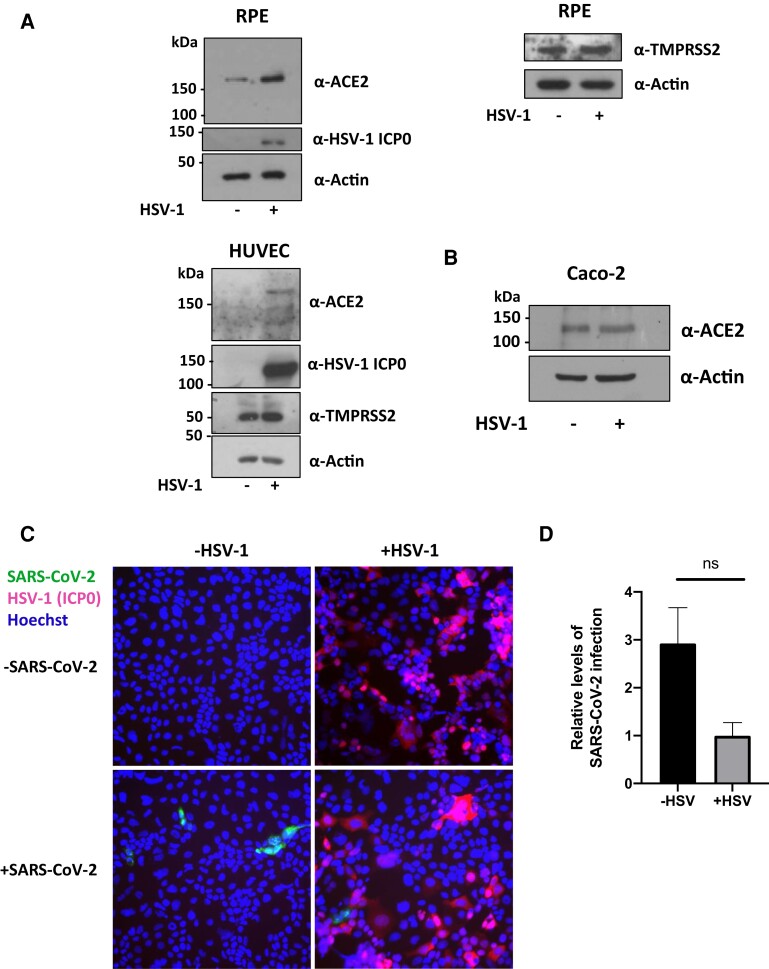
Prior HSV-1 infection does not increase SARS-CoV-2 infection in Caco-2 epithelial cells, although it can upregulate ACE2 in other cell types. *A*, RPE (above) or HUVEC (below) were infected with wild-type YFP-HSV-1 at an MOI of 1. At 24 hpi, protein lysates were harvested from cells and immunoblotted for ACE2, TMPRSS2, and the loading control, actin. *B*, Caco-2 cells were infected with wild-type YFP-HSV-1. At 24 hpi, protein was harvested and blotted for ACE2 levels. *C* and *D*, Caco-2 epithelial cells were infected with ΔgE HSV-1 for 24 hours before being infected with ZsGreen-tagged SARS-CoV-2 at an MOI of 0.01. *C*, Two days postinfection with SARS-CoV-2, cells were fixed, stained for HSV-1 ICP0 (red) and stained with Hoechst (blue), and imaged on a fluorescence microscope. *D,* Graph shows mean percent SARS-CoV-2 infection from randomly chosen images from 3 replicates. Error bars show standard error. Statistical significance was determined using a 2-tailed Mann-Whitney *U* test. Abbreviations: ACE2, angiotensin converting enzyme 2; hpi, hours postinfection; HSV, herpes simplex virus-1; HUVEC, human umbilical venous endothelial cells; MOI, multiplicity of infection; ns, not significant; RPE, retinal pigment epithelial cells; SARS-CoV-2, polymerase chain reaction.

Therefore, we infected RPE-1, HUVEC, and Caco-2 cells with YFP-tagged HSV-1 and harvested protein from these cells at 24 hpi to test whether, like HCMV, HSV-1 could upregulate ACE2. Figure 7*A* shows that HSV-1 infection did indeed upregulate ACE2 levels in RPE and HUVEC cells but failed to increase ACE2 levels in Caco-2 cells (Figure 7*B*). We then tested whether prior infection with HSV-1 could still increase SARS-CoV-2 infection in Caco-2 cells. To do this, Caco-2 cells were infected with ΔgE HSV-1 (ΔgE virus was used here, as HSV gE is a known Fc receptor which nonspecifically binds antibodies in immunofluorescence analyses), and 24 hpi, cells were superinfected with ZsGreen tagged SARS-CoV-2. After 2 days of SARS-CoV-2 infection, cells were fixed and stained for the HSV-1 ICP0 protein (an immediate early gene product). Starkly contrasting with HCMV, prior infection with HSV-1 seemed, instead, to inhibit SARS-CoV-2 superinfection (Figure 7*C* and 7*D*).

## DISCUSSION

The level of severity of SARS-CoV-2 infections varies drastically, and the reasons behind this are not yet well understood. Here, we have shown that lytic HCMV infection can augment SARS-CoV-2 infection in vitro by upregulating cellular ACE2. To our knowledge, only one other pathogen, influenza A virus, has been shown to act in a similar manner [3]. This effect of HCMV infection is of particular relevance to the ongoing pandemic: HCMV is extremely widespread, infecting 40%–90% of the population, and infection persists for life. Consequently, coinfections of HCMV with SARS-CoV-2 are likely common, and these results could have important consequences for the treatment of COVID-19. Interestingly, HCMV has a higher prevalence in certain ethnic minorities and those from areas of socioeconomic deprivation, and these individuals are also known to be disproportionately affected by severe SARS-CoV-2 infection [1]. Based on the results shown here, we speculate that HCMV infection could be one contributing factor in this disparity.

Our results indicate that productive HCMV infection increases SARS-CoV-2 infection in vitro. Although HCMV is carried as a latent infection for life, numerous studies, although not all [37, 38], have detected HCMV reactivation during COVID-19 infection, including hospital admitted patients with COVID-19–induced acute respiratory distress syndrome (ARDS) and COVID-19 patients on mechanical ventilation [8–15, 39–41]. This is perhaps not unexpected; HCMV reactivates sporadically in the lungs of healthy seropositive carriers [16], and additionally, HCMV reactivation frequencies are increased and less well controlled during inflammation, critical illness, ARDS, and mechanical ventilation. Indeed, one study found that the rates of HCMV reactivation in COVID-19 patients were even higher than in patients suffering from critical illness not caused by SARS-CoV-2 [12, 42]. Taken together, we think it likely that reactivation of HCMV in, for example, macrophages and increased dissemination into, for example, epithelial cells could result in secretion of factors that upregulate ACE2 and cause increased permissiveness of these cells for SARS-CoV-2 infection.

It remains unclear how and why HCMV infection upregulates ACE2, but we speculate that this may be a collateral effect of a factor secreted by HCMV-infected cells. For example, HCMV-infected cells are known to secrete IL-6, which has been shown to upregulate ACE2 in certain tissues [43]. Little has been published on the HCMV secretome in Caco-2 cells specifically and further work must be undertaken to identify this secreted factor. Although interferons have been reported to upregulate ACE2 [31], we found no evidence of this in Caco-2 cells. Moreover, pretreatment with IFN-β or IFN-λ has been shown to actually protect Caco-2 cells from subsequent SARS-CoV-2 infection, as might be expected, given the known antiviral nature of interferons [44]. Bai et al also found that prior treatment of Calu-3 cells with IFN-α inhibited SARS-CoV-2 infection [3]. Additionally, Busnadiego et al found that, although IFNs upregulated ACE2 mRNA, pretreatment of Calu-3s with IFN-β and -γ prior to infection with SARS-CoV-2 actually led to a severe drop in progeny virus titers, arguing that the antiviral effects of interferon outweighed any increase in ACE2 levels [33].

One limitation of our study is that we cannot definitively conclude that increased SARS-CoV-2 infection in the presence of HCMV was due to higher entry of SARS-CoV-2. The higher levels of SARS-CoV-2 infection in cells previously infected with HCMV were quantified 2 days after SARS-CoV-2 infection, which would have been more than enough time for SARS-CoV-2 to replicate and produce progeny virions capable of infecting other surrounding cells [24]. Therefore, it is also possible that HCMV drives higher levels of SARS-CoV-2 replication as well as increased entry of SARS-CoV-2. For example, it is known that SARS-CoV-2 infection routinely results in host cell death by proapoptotic signaling. However, HCMV, which has an extended life cycle (around 72 hours in some cell types), has evolved multiple mechanisms to protect cells from such stress-induced prodeath signaling. In doing so, it could potentially prolong the life of SARS-CoV-2–infected cells to, for example, allow more shedding of virus.

Our data also clearly show that HCMV upregulates ACE2 in HUVECs, a primary endothelial cell type that is commonly used in HCMV infection models. We also observed that HCMV mediated a drastic upregulation of TMPRSS2 in these cells. Endothelial cells are thought to be important influencers of disease in SARS-CoV-2 infections as they have a central role in controlling thrombosis and proinflammatory cytokine secretion and 2 of the major symptoms and causes of severe disease in COVID-19 are pervasive clotting and excessive inflammation [29, 45]. The involvement of endothelial cells in SARS-CoV-2–mediated pathology via direct infection is controversial, as multiple groups report that primary endothelial cells express little or no ACE2 (consistent with our analysis of HUVECs) and are poorly infected by SARS-CoV-2 [46–48]. Endothelial cell TMPRSS2 levels were also reported to be very low [48]. Consistent with this, infections of endothelial cells in vitro appear to be dependent on very high MOIs and often require overexpression of ACE2 on target cells [46–48]. However, some reports have detected the presence of virus in the endothelium of samples from patients infected with SARS-CoV-2 [28, 49]. The results shown here could provide a route for endothelial cell infection in vivo by SARS-CoV-2 despite normally low ACE2 and TMPRSS2 levels. Interestingly, influenza virus has been shown to elevate ACE2 levels on A549 lung epithelial cells to the point that they are now permissive for SARS-CoV-2 entry [3].

At least 2 reports have described a correlation between HCMV reactivation and longer stays on mechanical ventilation during COVID-19 [12, 41]. The results presented in this paper warrant further clinical investigation to determine if HCMV augments SARS-CoV-2 infection in vivo and whether antivirals to HCMV or possibly anti-CMV immunoglobulin [50] could ameliorate severe COVID-19 in HCMV-seropositive individuals.

## Supplementary Data

Supplementary materials are available at *The Journal of Infectious Diseases* online. Consisting of data provided by the authors to benefit the reader, the posted materials are not copyedited and are the sole responsibility of the authors, so questions or comments should be addressed to the corresponding author.

## Notes

***Consortia***. Members of the Cambridge Institute of Therapeutic Immunology and Infectious Disease-National Institute for Health Research (CITIID-NIHR) COVID BioResource Collaboration are: Principal investigators: Stephen Baker, John Bradley, Gordon Dougan, Christoph Hess, Ian Goodfellow, Ravi Gupta, Nathalie Kingston, Paul J. Lehner, Paul A. Lyons, Nicholas J. Matheson, Willem H. Owehand, Caroline Saunders, Kenneth G. C. Smith, Charlotte Summers, James E. D. Thaventhiran, Mark Toshner, and Michael P. Weekes. CRF and volunteer research nurses: Ashlea Bucke, Jo Calder, Laura Canna, Jason Domingo, Anne Elmer, Stewart Fuller, Julie Harris, Sarah Hewitt, Jane Kennet, Sherly Jose, Jenny Kourampa, Anne Meadows, Criona O’Brien, Jane Price, Cherry Publico, Rebecca Rastall, Carla Ribeiro, Jane Rowlands, Valentina Ruffolo, and Hugo Tordesillas. Sample logistics: Ben Bullman, Benjamin J. Dunmore, Stuart Fawke, Stefan Gräf, Josh Hodgson, Christopher Huang, Kelvin Hunter, Emma Jones, Ekaterina Legchenko, Cecilia Matara, Jennifer Martin, Ciara O’Donnell, Linda Pointon, Nicole Pond, Joy Shih, Rachel Sutcliffe, Tobias Tilly, Carmen Treacy, Zhen Tong, Jennifer Wood and Marta Wylot. Sample processing: Laura Bergamaschi, Ariana Betancourt, Georgie Bower, Aloka De Sa, Madeline Epping, Stuart Fawke, Oisin Huhn, Sarah Jackson, Isobel Jarvis, Jimmy Marsden, Francesca Nice, Georgina Okecha, Ommar Omarjee, Marianne Perera, Nathan Richoz, Rahul Sharma, and Lori Turner. Clinical data collection: Eckart M. D. D. De Bie, Katherine Bunclark, Masa Josipovic, Michael Mackay, Federica Mescia, Alice Michael, Sabrina Rossi, Mayurun Selvan, Sarah Spencer, and Cissy Yong. Royal Papworth Hospital ICU: Ali Ansaripour, Alice Michael, Lucy Mwaura, Caroline Patterson, and Gary Polwarth. Addenbrooke’s Hospital ICU: Petra Polgarova and Giovanni di Stefano. NIHR BioResource: John Allison, Heather Biggs, Helen Butcher, Daniela Caputo, Matt Chandler, Patrick F. Chinnery, Debbie Clapham-Riley, Anne-Maree Dean, Eleanor Dewhurst, Christian Fernandez, Anita Furlong, Anne George, Barbara Graves, Jennifer Gray, Sabine Hein, Tasmin Ivers, Mary Kasanicki, Emma Le Gresley, Rachel Linger, Sarah Meloy, Alexei Moulton, Francesca Muldoon, Nigel Ovington, Sofia Papadia, Roxana Paraschiv, Christopher Penkett, Isabel Phelan, Venkatesh Ranganath, Jennifer Sambrook, Katherine Schon, Hannah Stark, Kathleen E. Stirrups, Paul Townsend, Julie von Ziegenweidt, Neil Walker, and Jennifer Webster.

***Acknowledgment.*** We thank Paula Rayner for technical assistance.

***Disclaimer.*** The funder had no role in study design, data collection, analysis, decision to publish, or writing of the manuscript.

***Financial support.*** This work was supported by the Medical Research Council (grant numbers MR/S00081X/1 Programme Grant and RG86932 Doctoral Training Grant).

## Supplementary Material

jiac452_Supplementary_Data
