# Supplementary material for: Human Cytomegalovirus Infection of Epithelial Cells Increases SARS-CoV-2 Superinfection by Upregulating the ACE2 Receptor
Source: J Infect Dis. 2022 Nov 21;227(4):543–53. doi: 10.1093/infdis/jiac452 (PMC9927080; doi:10.1093/infdis/jiac452)
Supplement: jiac452_Supplementary_Data [file jiac452_supplementary_data.docx]

Supplementary Figure 1

B)


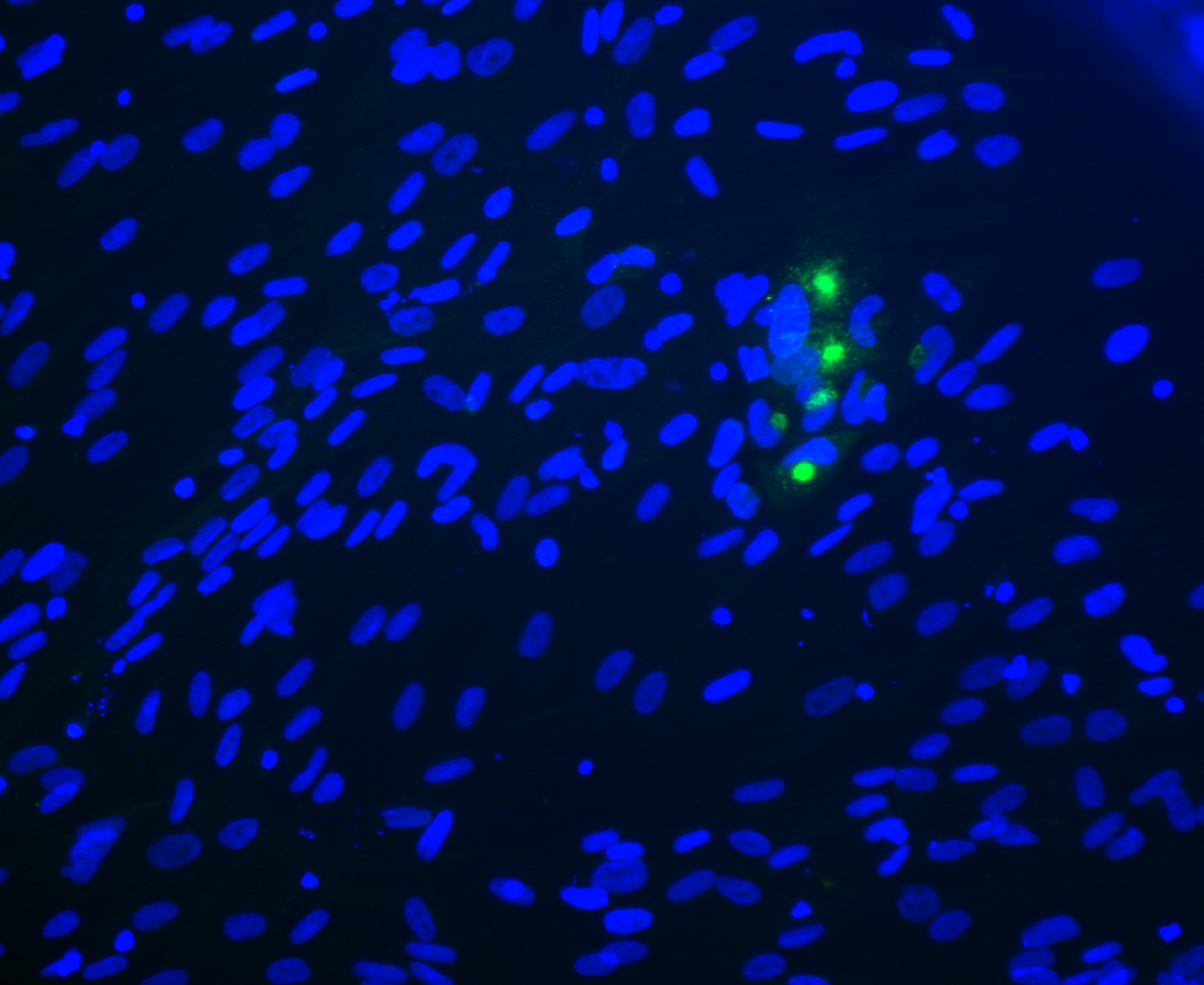

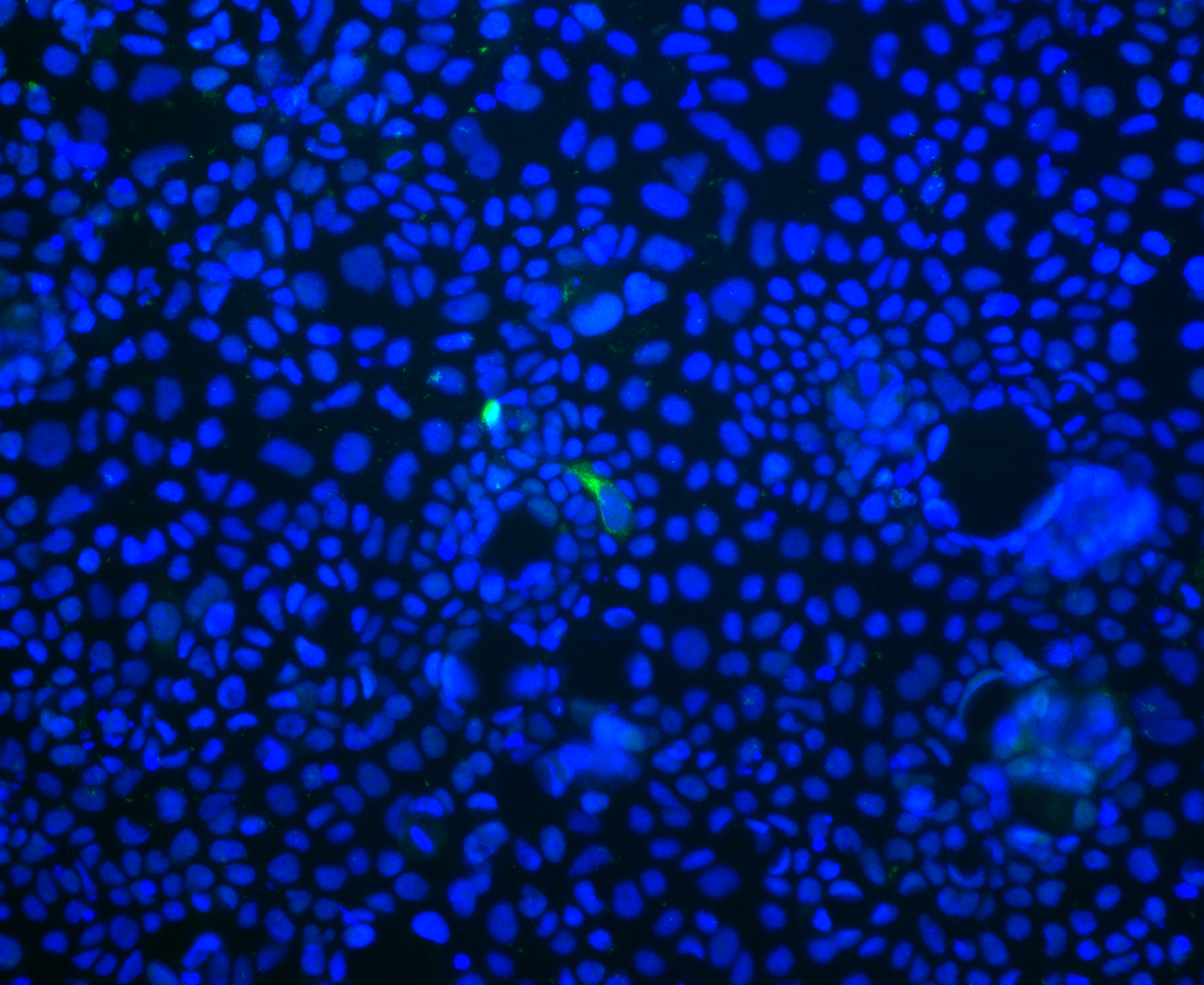


A)

α-pp28

Hoechst

α-pp28

Hoechst

**Figure S1. Caco-2 cells support productive HCMV infection. A)** Caco-2 cells were infected with HCMV at an MOI of 1. Cells were fixed and stained for the late lytic protein, pp28, at 5 d.p.i and imaged on a fluorescence microscope. **B)** Supernatant from Caco-2 cells infected with HCMV at an MOI of 1 was harvested at 4 d.p.i. Supernatants were then transferred to uninfected human foreskin fibroblasts and incubated for 6 days, after which they were fixed and stained for pp28. Plaques formed are indicative of infectious virus present in the supernatant.
